# Comparison of Kristjansson Respiratory Score and Wang Respiratory Score in infants with bronchiolitis in a hospital emergency department

**DOI:** 10.1142/S1013702520500146

**Published:** 2020-08-21

**Authors:** Frederico Ramos Pinto, Liane Correia-Costa, Inês Azevedo

**Affiliations:** 1 Department of Physical Medicine and Rehabilitation, Centro Hospitalar Universitário São João, Alameda Professor Hernâni Monteiro, 4202-451 Porto, Portugal; 2 Instituto de Ciências Biomédicas Abel Salazar, Universidade do Porto, Porto, Portugal; 3 EPIUnit - Instituto de Saúde Pública, Universidade do Porto, Porto, Portugal; 4 Division of Pediatric Nephrology, Centro Materno-Infantil do Norte, Centro Hospitalar Universitário do Porto, Porto, Portugal; 5 Department of Obstetrics-Gynecology and Pediatrics, Faculty of Medicine, Universidade do Porto, Alameda Professor Hernâni Monteiro, 4200-319, Porto, Portugal; 6 Department of Pediatrics, Centro Materno-Pediátrico, Centro Hospitalar Universitário São João, Alameda Professor Hernâni Monteiro, 4202-451 Porto, Portugal; frederico.ramospinto@gmail.com

**Keywords:** Bronchiolitis, infants, symptom assessment, severity score

## Abstract

**Objective:**

Several respiratory scores have been created to evaluate bronchiolitis’ severity level, but it is still not clear which is the best score. The aim of this study is to compare the Wang Respiratory Score (WRS) and the Kristjansson Respiratory Score (KRS) in the setting of an emergency room.

**Methods:**

We performed a prospective observational study with 60 infants with bronchiolitis admitted to a paediatric emergency department. For both scores, we assessed inter-rater reliability between two different health professionals (physician and physiotherapist), internal consistency, and correlation with SpO_2_ testing the intraclass-correlation coefficient (ICC), weighted kappa, Cronbach α coefficient and Spearman tests, respectively.

**Results:**

The inter-rater reliability was higher in KRS (ICC 0.79) and the Cronbach α and weighted kappa had similar values in KRS versus WRS. The correlation between the KRS/WRS and SpO_2_ was poor/moderate upon admission and discharge for the first observer and the second observer.

**Conclusions:**

While the internal consistency was similar in both scores, inter-rater reliability of KRS was higher than WRS, which allows us to conclude that it would have more consistent results when used to assess bronchiolitis’ level of severity by health personnel in a busy hospital emergency room.

## Introduction and Objectives

Acute bronchiolitis, an inflammation of the lower respiratory tract, is one of the most common respiratory diseases affecting infants and it imposes an enormous burden on healthcare resources worldwide.^1^ In most cases, however, it is a self-limited condition and one that can be treated at home.^1,2,3^ In fact, only 1–3% of children need to be hospitalized.^4,5^ Aspects such as clinical history, respiratory parameters, risk factors and oxygen saturation levels, are used by physicians in emergency departments to distinguish between a mild, moderate or severe case of bronchiolitis, in order to decide if hospitalization is necessary.^3^

An accurate assessment of the level of severity is thus crucial in the case of bronchiolitis for two main reasons: (a) to decide on the need for close monitoring and hospitalization; and (b) for research purposes, to allow comparisons between study groups. Several respiratory scores have been created to assess the level of severity and progression of the disease as well as the efficacy of therapeutic interventions. Examples of such scores are the Tal Score,^6^ the Modified Tal Score (MTS),^7^ the Respiratory Distress Assessment Instrument (RDAI),^8^ the Wang Respiratory Score (WRS)^9^ and the Kristjansson Respiratory Score (KRS).^10^ Most of these scores use the main clinical signs of bronchiolitis for assessment of severity level, not being clear which is the best score and which should be used in a given setting.

Currently, the RDAI is the score most commonly used by physicians in a clinical trial setting, but although it has adequate inter-rater reliability, the construct validity ranges from poor to moderate. In addition, its use by health professionals can be challenging,^11^ as it is difficult to evaluate the presence of wheezing throughout the respiratory cycle at high respiratory rates.

In the context of this study, we have decided to examine WRS^9^ and KRS,^10^ scores specifically developed to assess bronchiolitis’ severity level. The motivations behind this choice were threefold: (a) the acknowledged need for a score easy to use in a hospital emergency by health professionals other than physicians; (b) the advantage of being easy to apply in emergency departments; and (c) the fact that these scores had been designed on the basis of specific physical parameters common in children with acute lower airway infections, such respiratory frequency, chest recessions and wheezing.^12,13^

Both scores are very similar in terms of the parameters they include (the difference being that KRS additionally includes ‘skin color’), and had never been studied/used by different health professions. Chin and Seng^14^ study was the first which compared these two scores, but included only physicians. The purpose of this study is to compare WRS and Kristjansson Score and establish the inter-rater reliability and internal consistency when used by physicians and a physiotherapist.

## Materials and Methods

### Study design and population

This was a prospective observational study in a convenience sample of Portuguese infants admitted with acute bronchiolitis. Data were collected between January and May of 2010, in the paediatric emergency department of the Centro Hospitalar Universitário São João, Porto (CHUSJ), a tertiary care hospital. Inclusion criteria were: all children aged less than 24 months diagnosed with bronchiolitis by a physician from one of two different emergency teams working on Monday and Wednesday (from 4 p.m. to 2 a.m.) or Saturday and Sunday (from 9 a.m. to 2 a.m.) in weekend. Children with history of prematurity, underlying cardiopulmonary disease or immunodeficiency were not excluded from the study, since the aim of this study was to evaluate the performance of respiratory scores in defining the level of respiratory distress, regardless of the presence of risk factors. Exclusion criterions were as follows: all children with severe hypoxemia, needing oxygen supplementation or needing invasive or non-invasive ventilation. These are all criteria for inpatients with bronchiolitis and thus not compatible with this study focused on outpatients only.

### Assessment

Two observers (one physician and one physiotherapist) independently assessed all children using both WRS (Table A.5) and KRS (Table A.6) in the paediatric emergency department. This was completed upon admission and discharge within a time frame of approximately 15 min between observations. The first assessment was always made by the physician, being the physiotherapist the second before the medical treatment starts. Before the start of the study, all observers were briefed on the purpose of the study and how to fill in each respiratory score. The first observer (from a group of 24) was a physician, a paediatrician or a resident in paediatrics, and the second observer was one physiotherapist, the main investigator. The physician’s years of experience were not taken into account and their inter-rater reliability was based in the Chin and Seng^14^ study, when distributing the cases in the emergency department and each observer was blinded to the other observer’s assessment. The children were evaluated in a calm environment, while awake and not crying.

The WRS is a 4-item score which includes respiratory rate, wheezing, chest retraction and general condition. Each clinical sign is scored from zero to three except for the general condition, which is scored zero for normal, or three for irritability or lethargy. The total score ranges from 0 to 12.^9^ The KRS is a 5-item score which includes respiratory rate, chest recessions/retractions, breath sound/wheezing, skin color and general condition. Each clinical sign is scored from zero to two and the total score ranges from 0 to 10.^10^ Both scores establish severity as the total score increases.

The respiratory rate was determined by counting the number of breath cycles during 60 s. Chest recessions/retractions, skin color and general condition were assessed by observation and breath sounds/wheezing with a stethoscope.

The SpO_2_ was recorded in all children, while breathing room-air, with a pulse-oximeter (Dinamap DPC301N-PR, GE Medical Systems Information Technologies, Inc. Milwaukee, USA). The maximum SpO_2_ was determined both upon admission and discharge, after the pulse-oximeter had been branched for at least two minutes. If the SpO_2_ was below 92%, supplemental oxygen was provided to the patient.

For each patient, additional baseline data were collected, including demographic data, personal and family past medical history. This comprised of medical diagnosed food allergies, rhinitis, asthma and atopic dermatitis, exposure to tobacco smoke, contact with other children, parents schooling level, and medication administered in the emergency department, including supplemental oxygen.

This study was approved by the Ethics Committee of the CHUSJ, Porto. It also complied with the Helsinki Declaration and the current national legislation. Verbal and written consent was obtained from caregivers on behalf of all children enrolled in this study.

### Statistical analysis

Data were analyzed using IBM SPSS Statistics version 23.0. A Kolmogorov–Smirnov normality test was used and a p value of p=0.002 suggested strong non-normality, leading us to the use non-parametric tests to analyze the data. Skewed variables are presented as median and 25th and 75th percentiles. Differences between groups were evaluated using Mann–Whitney tests, for continuous variables, or Chi-square tests, for categorical variables. The internal consistency of scores items was evaluated by calculating the Cronbach α coefficient; values above 0.70 were considered to represent a good internal consistency.^15,16^ Inter-rater reliability of the scores between the first and the second observer were determined using weighted kappa for ordinal variables and intraclass-correlation coefficient (ICC) for continuous variables, on mean-rating (k=2), one-way random-effects model.^17,18^ Power of ICC was established *post hoc*. The idea of using weighted kappa is that disagreements involving distant values are weighted more heavily than disagreements involving more similar values. Agreement was considered to be almost perfect if k was greater than 0.80, substantial if within the range 0.61–0.80, moderate if within the range 0.41–0.60, fair if within the range 0.21–0.40, and slight if below 0.20.^18,19^ The correlation of total respiratory scores with SpO_2_ was determined using Spearman tests, considering ≤0.25 as little or no correlation, fair if within the range 0.25–0.50, moderate to good if within the range 0.50–0.75, and good to excellent if ≥0.75.^20^ A p value of less than 0.05 was considered statistically significant.

## Results

Sixty children were enrolled (median age of 6 (4–10) months) of which 51.7% had six months or less and 21 were male (54.5%). Ten children had a history of prematurity or presented a previous diagnosis of a cardiopulmonary disease. Moreover, 50% of the children were diagnosed with a first episode of bronchiolitis. The baseline characteristics of the sample, according to the age group (<= or >6 months of age), are depicted in Table A.1.

The median value of SpO_2_ was 96% (93–98) upon admission and 97% (95–99) at discharge. In the emergency department, 15% of children (n=9) were managed without specific therapeutic interventions while all the others were treated with bronchodilators, hypertonic saline, and/or oral steroids. Oxygen was prescribed in 3.3% of cases (n=2) (Table A.1). 75% (n=45) of the children were discharged from the emergency department (Table A.1).

The data collected through the WRS and KRS respiratory scores are detailed in Table A.2. The median (IQ) score of WRS was 5.5 (4–7) and 6 (5–9) upon admission, and 3 (2–5) and 5 (3–7) at discharge, for the first (physician) and second (physiotherapist) observer, respectively. The median score of KRS was 4 (3–5) and 4.5 (4–5) upon admission, and 3 (2–4) and 3 (3–5) at discharge, for the first and second observer, respectively.

There was a fair correlation between KRS/WRS and SpO_2_ upon admission and discharge for the first observer and the second observer (Table A.3). The inter-rater reliability was good for KRS (ICC 0.78) and moderate for WRS (ICC 0.69), with a power of 0.756 (Table A.3). Internal consistency of KRS was graded as sufficient with a Cronbach α ranging from 0.43 to 0.78 (Table A.4). The inter-rater reliability for individual clinical signs in KRS was similar to WRS, with weighted kappa ranging from 0.21 (0.04–0.39) to 0.50 (0.33–0.68), and the respiratory rate presented the highest reliability with a kappa value of 0.50 (0.33–0.68), followed by chest recession with 0.46 (0.23–0.64), skin color with 0.46 (0.13–0.78), breath sounds with 0.36 (0.15–0.57) and general condition 0.21 (0.04–0.39) (Table A.4).

## Discussion

In this study, we report a sufficient internal validity and good reliability of both scoring systems, when applied by physicians and a physiotherapist to assess the clinical severity of a child’s observed bronchiolitis in the setting of a busy emergency department. Both scores performed well with each item significantly contributing to the overall score. The correlation with SpO_2_ was fair in both scores and the inter-rater reliability obtained a correlation magnitude higher in the KRS than in the WRS.

The SpO_2_ determined by pulse oximeter is usually used by physicians to establish bronchiolitis severity, but should be considered alongside other factors such as respiratory frequency, heart rate, age and feeding intake to get the most accurate assessment of bronchiolitis level of severity.^3,21,22^ The average negative correlation between SpO_2_ and both respiratory scores obtained in our study was similar to that reported by Chin and Seng.^14^ The fair correlation could not be seen as a negative outcome because a large fluctuation of SpO_2_ is normal during a bronchiolitis episode and it is normal to observe low levels of SpO_2_ in light or moderate bronchiolitis.^22^ This could be the reason for the fair correlation with both scores.

Despite the fact that the physician’s level of experience varied considerably, the inter-rater agreement was found to be good. The respiratory rate was shown to be the best parameter to determine respiratory distress in both scores and for both observers, followed by breath sounds/wheezing in WRS and chest recessions in KRS. These findings were expected and other researchers have also previously reported that objective physical signs present a higher inter-rater agreement than subjective physical signs.^9,23^

General condition showed to be the least reliable parameter in both respiratory scores. This may be explained in part by the brief explanation of scores given to first observers and by the subjectivism of this parameter. In the case of KRS, observers had a footnote explaining that under ‘General Condition’, observers should assess the general complexion of children in addition to asking caretakers if they had noticed a food intake decrease or change in sleeping pattern. However, in the case of WRS, observers did not have this kind of guidance and could only score as zero or three upon a simple observation of children complexion at that moment. This might have led to doubt in cases with small decreases in nutrition intake or slight alteration in sleeping pattern — in most cases, these were recorded as a zero when they should have been recorded as 3.^9,10^

Our study did not aim to determine a model for predicting admission but to examine the inter-rater agreement of the scoring system when used by different health professionals with different levels of experience, not only for clinical purposes but also for research purposes. We can consider that this objective was accomplished as we reported an inter-rater reliability higher than 0.70, with KRS obtaining 0.78 and WRS 0.69.

One of the identified strengths of our study was the fact that among the participants not only children with a first episode of bronchiolitis were included, but also children with exclusion factors (e.g., cardiopulmonary disease, prematurity) allowing us a larger generalization in terms of population. Another positive aspect was the fact that the assessments between observers were all performed within 15 min of each other — this not only followed the practice in other studies^7,24^ and allowed comparison, but also reduced the probability of having the change in the children’s clinical condition interfering with the clinical score assignment.

Most of the previous comparative studies between two scores, or treatments efficacy studies using one specific score, were conducted in inpatients and only few studies have been conducted in an emergency department environment and outpatients.^21,25,26,27^ This is probably due to the fact that it is easier to obtain a sample of hospitalized patients than that of outpatients, since it is unpredictable when eligible patients access the emergency department and are in conditions to be discharged home. In our study, although recognizing the challenges of recruiting patients in the emergency department, we specifically tried to evaluate the performance of respiratory scores used by different health professionals in an emergency department. In a hospital, this is after all the first point of call for patients with bronchiolitis and a very different setting from the regular ward, given the higher number of patients and medical personnel. It was thus a particularly positive result to find a good KRS inter-rater reliability and a sufficient internal consistency in this different setting.

Some of the limitations of our study were the sample size and the study design, which limited our capacity to assess other important properties of the scores, namely their construct validity in regard to decision to admit or discharge comparing with length of stay, and responsiveness. The number of physicians and the number of physiotherapists involved should be more balanced, given that in this study, the unbalanced distribution turned out to limit our ability to calculate a more accurate inter-rater reliability. The difference in number of physicians and physiotherapists also violated some Kappa calculation, leading to an overestimation of the results which should be taken as a reference only.

Finally, another limitation was the insufficient information briefed to physicians, with no previous contact with the respiratory scores — this has led to unforeseen difficulties which might explain some of the reported inconsistencies.

In a future larger study, the construct validity of KRS should be established to prove the utility of this score in an emergency department.

## Conclusions

Both respiratory scores and most of the physical signs showed high agreement between observers. In fact, both scores present similar results in regard to their internal consistency. However, given that inter-rater reliability was higher in KRS than in WRS, KRS seems more consistent for use by health personnel in the assessment of children with bronchiolitis in the setting of an emergency room.

## Acknowledgments

We would like to thank all the physicians of the Emergency Department at Centro Hospitalar Universitário São João, EPE who helped the researcher apply the respiratory scores, namely Joana Miranda, Glória Silva, Liane Costa, Rita Milheiro, João Barreira, Bonito Vitor, Sofia Águeda, Catarina Ferraz, Andreia Lopes, Mariana Rodrigues, Carla Dias, Antonieta Azeredo, Paula Guerra, Joana Rebelo, Ruben Rocha, Isabel Fonseca and for providing all the help in analyzing the data and performing the statistical analysis and Rui Viana with all the pertinent subjections as reviewer.

## Conflict of Interest

The authors declare no potential conflicts of interest with respect to the research, authorship, and/or publication of this paper.

## Funding/Support

There was no financial support for the study.

## Author Contributions

Frederico Ramos Pinto conceptualized and designed the study, analyzed the data and drafted the initial paper, and approved the final paper as submitted.

Inês Azevedo and Liane Correia-Costa conceptualized and designed the study, supervised data collection, participated in and supervised data analyses, reviewed and revised the paper, and approved the final paper as submitted.

## Figures and Tables

**Table A.1.** Baseline characteristics of participants and selected outcomes according to age groups.

	≤6 Months (n=31)	>6 Months (n=29)	p value
**Demographics characteristics**			
Gender, male, n (%)	21 (54.5)	19 (47.5)	0.004
**Clinical history**			
Prematurity, n (%)	3 (9.7)	2 (6.9)	>0.001
Cardiopulmonary disease, n (%)	1 (3.2)	4 (13.8)	>0.001
First episode of bronchiolitis, n (%)	20 (64.5)	13 (44.8)	0.577
**Oxygen saturation**			
At admission, median, IQR	97 (95–99)	96 (92–97)	0.088
At discharge, median, IQR	98 (95–100)	97 (95–98)	0.056
Hospital admission, n (%)	9 (29.0)	6 (20.7)	0.020

*Notes*: Values presented are n (%) or median (P25–P75); mo: months; Entr/Disch: entrance/discharge.

**Table A.2.** Total respiratory scores at admission and discharge, by observers.

		Admission	Discharge
WRS	1st Obs., median (IQR)	5.5 (4–7)	3.0 (2–5)
	2nd Obs., median (IQR)	6.0 (5–9)	5.0 (3–7)
KRS	1st Obs., median (IQR)	4.0 (3–5)	3.0 (2–4)
	2nd Obs., median (IQR)	4.5 (4–5)	3.0 (3–5)

*Notes*: WRS: Wang respiratory score; KRS: Kristjansson respiratory score; Obs.: observer.

**Table A.3.** Correlation of SpO_2_ with totals of WRS and KRS and Inter-rater reliability between the two observers.

	SpO_2_				
	1st Observer		2nd Observer		
	Admission	Discharge	Admission	Discharge	ICC
WRS	−0.299	−0.313	−0.295	−0.409	0.686 (p=0.001; 95% CI=0.475–0.812)
	(p=0.020)	(p=0.015)	(p=0.022)	(p=0.001)	
KRS	−0.397	−0.349	−0.324	−0.427	0.780 (p=0.001; 95% CI=0.633–0.868)
	(p=0.002)	(p=0.006)	(p=0.012)	(p=0.001)	

*Notes*: SpO_2_: Oxygen saturation; WRS: Wang Respiratory Score; KRS: Kristjansson Respiratory Score; ICC: Intraclass Correlation Coefficient.

**Table A.4.** Internal consistency and inter-rater reliability for individual clinical signs in WRS and KRS.

		Cronbach α values	Weighted kappa (95% CI)
WRS	Respiratory rate	0.78	0.52 (0.35–0.69)
	Chest retraction	0.76	0.39 (0.19–0.59)
	Wheezing	0.68	0.50 (0.33–0.66)
	General condition	0.43	0.23 (0.03–0.43)
KRS	Respiratory rate	0.69	0.50 (0.33–0.68)
	Chest recession	0.65	0.46 (0.23–0.64)
	Breath sounds	0.70	0.36 (0.15–0.57)
	General condition	0.53	0.21 (0.04–0.39)
	Skin color	0.69	0.46 (0.13–0.78)

*Notes*: WRS: Wang Respiratory Score and KRS: Kristjansson Respiratory Score.

**Table A.5.** Wang respiratory score.

Score	0	1	2	3
Respiratory Rate (breaths/minute)	<30	30–45	46–60	>60
Wheezing	None	Terminal expiration or only with stethoscope	Entire expiration or audible on expiration without stethoscope	Inspiration and expiration without stethoscope
Retraction	None	Intercostal recession	Trachea-sternal recession	Severe with nasal flow
General Condition	Normal			Irritable/lethargic/poor feeding

**Table A.6.** Kristjansson respiratory score.

Score	0	1	2
Respiratory Rate (breaths/minute)	<40	40–60	>60
Chest Recession	None	Moderate (costodiaphragmatic)	Severe (as in 1 plus rib & jugular retraction)
Breath Sound	Vesicular	Wheeze +∕− rhonchi/rale	Severe wheeze +∕− rhonchi/rale
Skin Color	Normal	Pallor	Cyanosis
*General Condition	Not affected	Moderately affected	Severely affected

*Notes*: *(a) Not affected if activity and feeding is normal; (b) moderately affected if activity and feeding is less than normal and (c) severely affected if child looks ill and feeds poorly.
